# Spatial heterogeneity can lead to substantial local variations in COVID-19 timing and severity

**DOI:** 10.1073/pnas.2011656117

**Published:** 2020-09-10

**Authors:** Loring J. Thomas, Peng Huang, Fan Yin, Xiaoshuang Iris Luo, Zack W. Almquist, John R. Hipp, Carter T. Butts

**Affiliations:** ^a^Department of Sociology, University of California, Irvine, CA, 92697;; ^b^Department of Statistics, University of California, Irvine, CA, 92697;; ^c^Department of Criminology, Law, and Society, University of California, Irvine, CA, 92697;; ^d^Department of Sociology, Center for Studies in Demography and Ecology, Center for Statistics and Social Sciences, eScience, University of Washington, Seattle, WA, 98195;; ^e^Department of Computer Science, University of California, Irvine, CA, 92697;; ^f^Department of Electrical Engineering and Computer Science, University of California, Irvine, CA, 92697

**Keywords:** COVID-19, spatial heterogeneity, diffusion, health disparities, social networks

## Abstract

Standard epidemiological models for COVID-19 employ variants of compartment (SIR or susceptible–infectious–recovered) models at local scales, implicitly assuming spatially uniform local mixing. Here, we examine the effect of employing more geographically detailed diffusion models based on known spatial features of interpersonal networks, most particularly the presence of a long-tailed but monotone decline in the probability of interaction with distance, on disease diffusion. Based on simulations of unrestricted COVID-19 diffusion in 19 US cities, we conclude that heterogeneity in population distribution can have large impacts on local pandemic timing and severity, even when aggregate behavior at larger scales mirrors a classic SIR-like pattern. Impacts observed include severe local outbreaks with long lag time relative to the aggregate infection curve, and the presence of numerous areas whose disease trajectories correlate poorly with those of neighboring areas. A simple catchment model for hospital demand illustrates potential implications for health care utilization, with substantial disparities in the timing and extremity of impacts even without distancing interventions. Likewise, analysis of social exposure to others who are morbid or deceased shows considerable variation in how the epidemic can appear to individuals on the ground, potentially affecting risk assessment and compliance with mitigation measures. These results demonstrate the potential for spatial network structure to generate highly nonuniform diffusion behavior even at the scale of cities, and suggest the importance of incorporating such structure when designing models to inform health care planning, predict community outcomes, or identify potential disparities.

## Significance

We examine the effects of an uneven population distribution on the spread of the COVID-19 disease spread, using a diffusion model based on interpersonal contact networks. Taking into account spatial heterogeneity, the spread of COVID-19 is much “burstier” than in standard epidemiological models, with substantial local disparities in timing and severity and long lags between local outbreaks. We show that spatial heterogeneity may produce dramatic differences in social exposures to those with the illness, and may stress health care delivery systems in ways that are not well captured by standard SIR-like models.

Since its emergence at the end of 2019, severe acute respiratory syndrome coronavirus 2 (SARS-CoV-2) has spread rapidly to all portions of the globe, infecting over 20 million people as of mid-August 2020 (1). The disease caused by this virus, denoted COVID-19, generally manifests as a respiratory illness that is spread primarily via airborne droplets. While most cases of COVID-19 are nonfatal, a significant fraction of those infected require extensive supportive care, and the mortality rate is substantially higher than more common infectious diseases such as seasonal influenza (2). Even for survivors, infection can lead to long-term damage to the lungs and other organs, leading to long convalescence times and enhanced risk of secondary complications (3, 4). By early March of 2020, COVID-19 outbreaks had appeared on almost every continent, including significant clusters within many cities (5). In the absence of an effective vaccine, public health measures to counteract the pandemic in developed nations have focused on social distancing measures that seek to slow diffusion sufficiently to avoid catastrophic failure of the health care delivery system. Both the planning and public acceptance of such measures have been highly dependent upon the use of epidemiological models to probe the potential impact of distancing interventions, and to anticipate when such measures may be loosened with an acceptable level of public risk. As such, the assumptions and behavior of COVID-19 diffusion models are of significant concern.

Currently, dominant approaches to COVID-19 modeling (6–8) are based on compartment models (often called SIR models, after the conventional division of the population into susceptible, infected, and recovered groups in the most basic implementations) that implicitly treat individuals within a population as geographically well mixed. While some such models include differential contact by demographic groups (e.g., age), and may treat states, counties, or, occasionally, cities as distinct units (e.g., work by ref. 9), those models presently in wide use do not incorporate spatial heterogeneity at local scales (e.g., within cities). Past work, however, has shown evidence of substantial heterogeneity in social relationships at regional, urban, and suburban scales (10–12), with these variations in social network structure impacting outcomes as diverse as regional identification (13), disease spread (14), crime rates (15), neighborhood identification, and development (12, 16). If individuals are not socially “well-mixed” at local scales, then it is plausible that diffusion of SARS-CoV-2 via interpersonal contacts will likewise depart from the uniform mixing characteristic of the SIR models. Indeed, at least one computational study (17) using a fairly “generic” (non-COVID) diffusion process on realistic urban networks has shown considerable nonuniformity in diffusion times, suggesting that such effects could hypothetically be present. Variations across local regions on the pandemic timing, severity, and the hospital load could have huge impacts on the social outcomes of different population groups (e.g., racial/ethnic groups) in the pandemic, given the heterogeneity of their spatial distribution in urban and suburban areas (18, 19). However, it could also be hypothesized that such effects would be small perturbations to the broader infection curve captured by conventional compartment models, with little practical importance. The question of whether these effects are likely to be present for COVID-19, and, if so, their strength and size, has, to date, remained open.

In this paper, we examine the potential impact of local spatial heterogeneity on COVID-19, modeling the diffusion of SARS-CoV-2 in populations whose contacts are based on spatially plausible network structures. We focus here on the urban context, examining 19 different cities in the United States. We simulate the population of each city in detail (i.e., at the individual level), simulating hypothetical outbreaks on the contact network in each city in the absence of measures such as social distancing. Despite allowing the population to be well mixed in all other respects (i.e., not imposing mixing constraints based on demographic or other characteristics), we find that spatial heterogeneity alone is sufficient to induce substantial departures from spatially homogeneous SIR behavior. Among the phenomena observed are “long lag” outbreaks that appear in previously unharmed communities after the aggregate infection wave has largely subsided, frequently low correlations between infection timing in spatially adjacent communities, and distinct subpatterns of outbreaks found in some urban areas that are uncorrelated with the broader infection pattern. Gaps between infection peaks at the intraurban level can be large, for example, on the order of weeks or months in extreme cases, even for communities that are within kilometers of each other. Such heterogeneity is potentially consequential for the management of health care delivery services: As we show, using a simple “catchment” model of hospital demand, local variations in infection timing can easily overload hospitals in some areas, generating “hospital deserts” (20), while leaving others relatively empty (absent active reallocation of patients). Likewise, we show that individuals’ social exposures to others who are morbid or deceased vary greatly over the course of the pandemic, potentially leading to differences in risk assessment and bereavement burden for persons residing in different locations. Differences in outbreak timing and severity may exacerbate health disparities (since, e.g., surge capacity varies by community) and may even affect perception of and support for prophylactic behaviors among the population at large, with those in so-far untouched communities falsely assuming that the pandemic threat is either past or was exaggerated to begin with, or attributing natural variation in disease timing to the impact of health interventions.

We note at the outset that the models used here are intended to probe the hypothetical impact of spatial heterogeneity on COVID-19 diffusion within particular scenarios, rather than to produce high-accuracy predictions or forecasts. For the latter applications, it is desirable to incorporate many additional features that are here simplified to facilitate insight into the phenomenon of central interest. In particular, we do not incorporate either demographic effects or social distancing (21, 22), allowing us to consider a setting that is as well mixed as possible (and hence as close as possible to an idealized SIR model), with the exception of spatial heterogeneity. As we show, even this basic scenario is sufficient to produce large deviations from the SIR model. Despite the simplicity of our models, we do note that the approach employed here could be integrated with other factors and calibrated to produce models intended for forecasting or similar applications.

## Materials and Methods

### Spatial Network Data.

Networks are generated using population distributions from the most recent US Census in 2010. Network construction followed the same methodology as Butts et al. (23). Hospital information was obtained from the Homeland Infrastructure Foundation-Level Data (HIFLD) database (24). HIFLD is an initiative that collects geospatial information on critical infrastructure across multiple levels of government. We employ the national-level hospital facility database, which contains locations of hospitals for the 50 US states; Washington, DC; and US territories of Puerto Rico, Guam, American Samoa, Northern Mariana Islands, Palau, and Virgin Islands; underlying data are collated from various state departments or federal sources (e.g., Oak Ridge National Laboratory). We employ all hospitals within our 19 target cities, excluding facilities closed since 2019. Latitude/longitude coordinates and capacity information were employed to create a spatial database that includes information on the number of beds in each hospital. The capacity information includes the number of beds that each hospital has available, and can be used to assess strain that a surge in hospitalizations could create.

The dates of the first confirmed case and all of the death cases for King County, where Seattle is located, were obtained from *The New York Times*, based on reports from state and local health agencies (25). The death rate was calculated based on population size of each county from the 2018 American Community Survey, and employed to calibrate the infection rate (the only free parameter in the models used here); details are provided in *SI Appendix*.

We ran 10 replicates of the COVID-19 diffusion process in each of our 19 cities, seeding with 25 randomly selected infections in each replicate and following the course of the diffusion until no infectious individuals remained. Simulations were performed using a combination of custom scripts for the R statistical computing system (26) and the statnet library (27–29). Analyses were performed using R.

### Methods.

COVID-19 is typically transmitted via direct contact with infected individuals, with the greatest risk occurring when an uninfected person is within approximately six feet of an infected person for an extended period. Such interactions can be modeled as events within a social network, where individuals are tied to those with whom they have a high hazard of intensive interaction. In prior work, this approach has been successfully employed for modeling infectious diseases ranging from HIV (30) and influenza (31) to Zika (32). To model networks of potential contacts at scale, we employ spatial network models (33), which are both computationally tractable and able to capture the effects of geography and population heterogeneity on network structure (23). Such models have been successfully used to capture social phenomena ranging from neighborhood-level variation in crime rates (15) and regional identification (13) to the flow of information among homeless persons (34).

The spatial network models used here allow for complex social dependence through a kernel function, referred to as the *social interaction function* (SIF). The SIF formally defines the relationship between two individuals based on spatial proximity. For example it has been shown that many social interaction patterns obey the Zipf law (35), where individuals are more likely to interact with others close by rather than far away [a pattern that holds even for online interactions (10)]. Here, we use this approach to model a network that represents the combination of frequent interactions due to ongoing social ties and contacts resulting from frequent incidental encounters (e.g., interactions with neighbors and community members).

We follow the protocol of refs. 15 and 23 to simulate social network data that combine the actual distribution of residents in a city with a prespecified SIF. We employ the model of ref. 15 with decennial Census data to produce large-scale social networks for 19 cities and counties in the United States—providing a representation of major urban areas in the United States (*SI Appendix*). Given these simulated networks, we then implement an individual-level SIR-like framework to examine COVID-19 diffusion. At each moment in time, each individual can be in a susceptible, infected but not infectious, infectious, deceased, or recovered state. The disease diffuses through the contact network, with currently infectious individuals infecting susceptible neighbors as a continuous time Poisson process with a rate estimated from mortality data (*SI Appendix*); recovered or deceased individuals are not considered infectious for modeling purposes. Upon infection, an individual’s transitions between subsequent states (and into mortality or recovery) are governed by waiting time distributions based on epidemiological data as described in *SI Appendix*. To begin each simulated trajectory, we randomly infect 25 individuals, with all others being considered susceptible. Simulation proceeds until no infectious individuals remain.

From the simulated trajectory data, we produce several metrics to assess spatial heterogeneity in disease outcomes. First, we present infection curves for illustrative cities, showing the detailed progress of the infection and its difference from what an SIR model would posit. We also present choropleth maps showing spatial variation in peak infection times, as well as the correlations between the infection trajectory within local areal units and the aggregate infection trajectory for the city as a whole. While an SIR model would predict an absence of systematic variation in the infection curves or the peak infection day for different areal units in the same city, geographically realistic models show considerable disparities in infection progress from one neighborhood to another. To quantify the degree of heterogeneity more broadly, we examine spatial variation in outcomes for each of our city networks. We show that large variations in peak infection days across tracts are typical (often spanning weeks or even months), and that overall correlations of within-tract infection trajectories with the aggregate urban trajectory are generally modest (a substantial departure from what would be expected from an SIR model).

In addition to these relatively abstract metrics, we also examine a simple measure of the potential load on the health care system in each city. Given the locations of each hospital in each city, we attribute infections to each hospital using a Voronoi tessellation (i.e., under the simple model that individuals are most likely to be taken to the nearest hospital if they become seriously ill). Examination of the potential hospital demand over time shows substantial differences in load, with some hospitals severely impacted while others have few cases. Finally, we consider the *social exposure* of individuals to COVID-19, by computing the fraction of individuals with a personal contact who is respectively morbid or deceased. Our model shows considerable differences in these metrics over time, revealing that the pandemic can appear very different to those “on the ground”—evaluating its progress by its impact on their own personal contacts—than what would be suggested by aggregate statistics.

## Results

### Smooth Aggregate Infection Trajectories Can Mask Local Outbreak Dynamics.

**Fig. 1.**
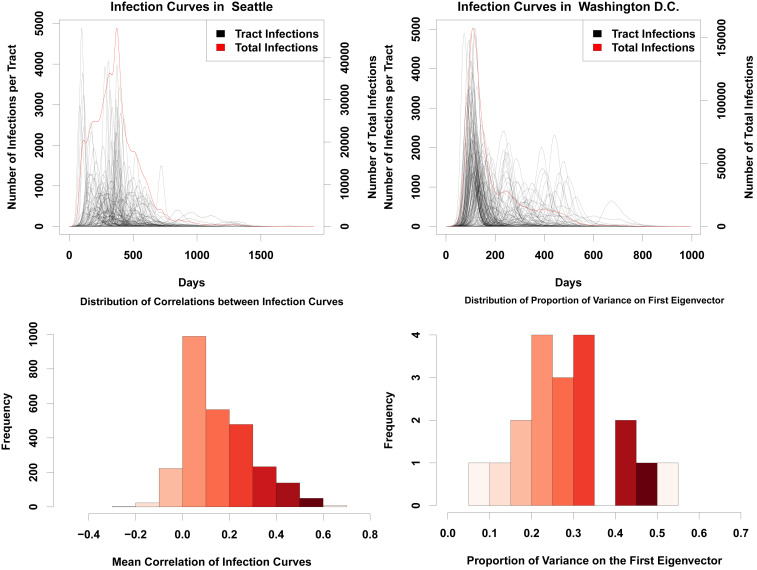
(*Top Left*) Infection curves for Seattle, WA. The red line is the curve for the whole city, while the black lines are the infection curves for each tract in the city. While the red curve is relatively smooth, this smoothness hides a significant amount of heterogeneity in the timing of the infection curves for each census tract. (*Top Right*) Infection curves for Washington, DC. As with Seattle, the city-level curve conceals considerable spatial variability in the infection’s progress. (*Bottom Left*) Histogram showing the mean pairwise correlation of infection curves for each tract within each city, across our entire sample. The infection curve in any given tract is likely to have a correlation of only around 0.2 with any other tract in the city. This histogram includes a single data point for each tract in the sample. (*Bottom Right*) Histogram of variance accounted for by the principal component of the standardized tract-level curve set. None of the principal components account for more than 60% of the variance, with most accounting for around only 35% of the total variance. The data points included here include a single amount of variance explained for each city.

When taken over even moderately sized regions, aggregate infection curves can appear relatively smooth. Although this suggests homogeneous mixing (as assumed, e.g., by standard SIR models), appearances can be deceiving. Fig. 1 shows typical realizations of infection curves for two cities (Seattle, WA, and Washington, DC), showing both the aggregate trajectory (red) and trajectories within individual Census tracts (black). While the infection curves in both cases are relatively smooth, and suggestive of a fairly simple process involving a sharp early onset followed by an initially sharp but mildly slowing decline in infections, within-tract trajectories tell a different story. Instead of one common curve, we see that tracts vary wildly in onset time and curve width, with some tracts showing peaks weeks or months after the initial aggregate spike has passed.

The cases of Fig. 1 are emblematic of a more systematic phenomenon: The progress of the infection within any given areal unit often has relatively little relationship to its progress in the city as a whole. Fig. 1, *Bottom* assesses this phenomenon over our entire sample, using two different consensus metrics. First, we simply compute the correlation between the infection curves in each pair of tracts (assessed at daily resolution), taking the mean for each tract of its correlation with all other tracts within the city; if the progress of the infection were uniform across the city, the mean correlations would be large and positive. Second, we provide a more direct assessment of the extent to which the set of infection curves can be summarized by a common pattern by taking the variance on the first principal component of the correlation matrix generated from the tract-level correlations discussed immediately above. As before, where different parts of the city experience similar patterns of growth and decline in infections, we expect the dimension of greatest shared variance to account for the overwhelming majority of variation in infection rates. Contrary to these expectations, however, Fig. 1 shows that there is little coherence in tract-level infection patterns. Mean correlations of local infection curves across tracts typically range from ∼0 to 0.5, with a mean of approximately 0.2, indicating very little correspondence between infection timing in one tract and that of another. The principal component analysis tells a similar story: Overall, we see that the first component accounts for relatively little of the total variance in trajectories, with, on average, only around 35% of variation in infection curves lying on the first principal component (and no observed case of the first component accounting for more than 60% of the variance). Interestingly, this variation is not explained by time required for the diffusion process to reach each tract (*SI Appendix*, Fig. S4), in contrast to the hypothesized importance of similar delays in a cross-national context (9). This confirms that local infection curves are consistently distinct, with behavior that is only weakly related to infections in the city as a whole. This is a substantially different scenario than what is commonly assumed in traditional SIR models.

### Peak Infection Days Can Vary Substantially, Even among Nearby Regions.

**Fig. 2.**
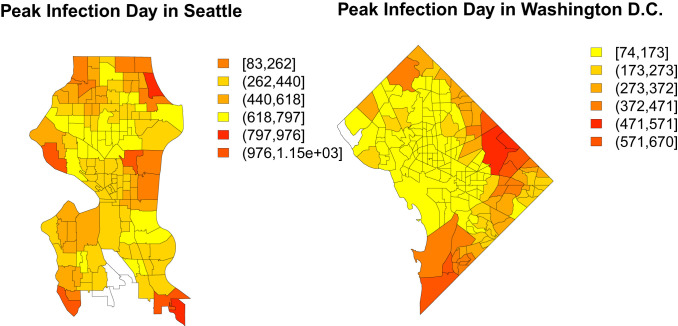
(*Left*) Choropleth showing the peak day of infection in each tract in the city of Seattle. The map shows significant variability in peak times, with nearby regions sometimes having sharply different patterns. In outlying parts of Seattle, the infection does not peak until almost a year past the first infections, while, in the more eastern and central parts of the city, the infection peaks much earlier. (*Right*) Times to peak infection for Washington, DC tracts. The southern and eastern part of the city has infections that are more delayed than in the central and northern parts of the city. Both of these maps show that there is a high degree of spatial heterogeneity present in the infection curves.

These differences in local infection curves are a consequence of the unevenness of the “social fabric” that spans the city: While the disease can spread rapidly within regions of high local connectivity, it can easily become stalled upon reaching the boundaries of these regions. Further transmission requires that a successful infection event occur via a bridging tie, an event with a potentially long waiting time. Such delays create potential opportunities for public health interventions (trace/isolate/treat strategies), but they can also create a false sense of security for those on the opposite side of the bridge (who may incorrectly assume that their area was passed over by the infection). Indeed, examining the time to peak infection across the cities of Seattle and Washington, DC (Fig. 2) shows that, while peak times are visibly autocorrelated, tracts with different peak times frequently border each other. Residents on opposite sides of the divide may be exposed to very different local infection curves, making risk assessment difficult.

**Fig. 3.**
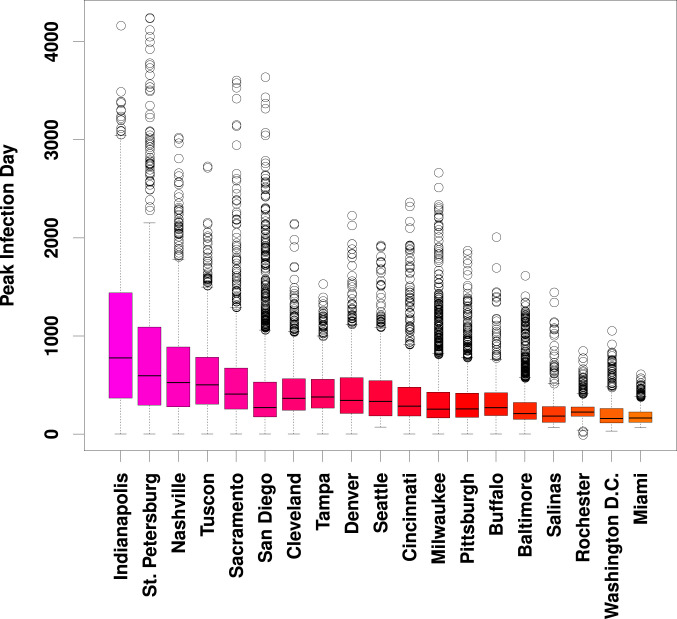
Marginal distributions of days to peak infection by tract, across 10 simulated trajectories. Although locales vary both in terms of overall median peak time and range of tract-level variation, large differences in peak time are nearly ubiquitous. (Trajectory specific distributions are shown in Fig. S2.)

The cases of Seattle and Washington, DC are not anomalous. Looking across multiple trajectories over our entire sample, Fig. 3 shows consistently high variation in per-tract peak infection times for nearly all study communities. (This variation is also seen within individual trajectories, as shown in *SI Appendix*, Fig. S2.) Although peak times in some cities are concentrated within an interval of several days to a week, it is more common for peak times to vary by several months or longer. Extreme delays in peak time arise from “slow burn” trajectories associated with very long infection chains, in which sequential transmission to only one or two alters at a time can sustain infection for greatly extended periods. Such gaps can arise naturally in inhomogeneous networks, but are far from what would be expected under uniform local mixing.

### Heterogeneous Impact Timing May Affect Hospital Load.

**Fig. 4.**
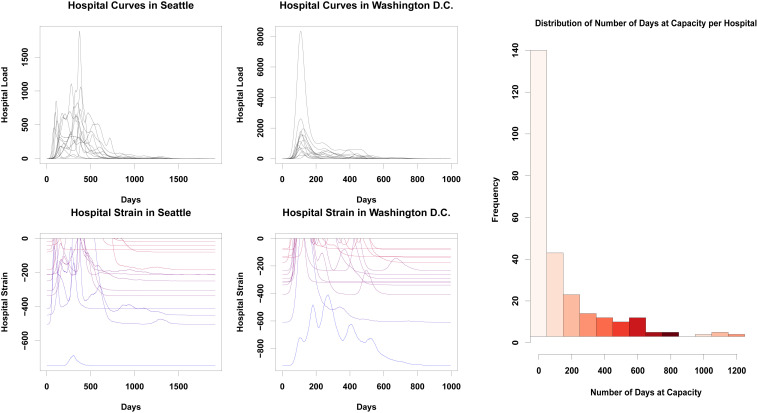
(*Top Left*) Numbers of infections attributed to each hospital in the city of Seattle, with each curve representing a different hospital. Hospital peak demand times vary markedly, with some getting the majority of their hospitalizations before day 100, and others peaking almost a year into the pandemic. (*Top Middle*) Hospitalizations in Washington, DC. As in Seattle, each hospital has a unique demand trajectory, with some hospitals not getting their peak of infections until more than a year after the infection begins. (*Bottom Left*) Hospital strain in Seattle, WA. Values closer to zero indicate that hospitals are more strained and have fewer open beds, while lower values suggest more resources are available; color varies from blue (low average strain) to red (high average strain). Much like the number of infections, there is a high degree of heterogeneity present here, with hospitals freeing up resources at different points across the first year of the pandemic. (*Bottom Middle*) Hospital strain for Washington, DC. Most hospitals get overwhelmed in the first 25 days of the pandemic, but then are able to recover at different times, usually within the second hundred days of the pandemic; some, however, are hit hard by a second wave, and others remain overwhelmed for several months. (*Right*) Marginal distribution of number of days without available beds, for all hospitals in our sample. While most hospitals will have only brief periods of overload, some will be at or over capacity for the entire pandemic, potentially several years.

Variation in the timing of COVID-19 impacts across the urban landscape has potential ramifications for health care delivery, creating unequally distributed loads that overburden some providers while leaving others with excess resources. To obtain a sense of how spatial heterogeneity in the infection curve could potentially impact hospitals, we employ a simple “catchment” model in which seriously ill patients are taken to the nearest hospital, subsequently recovering and/or dying as assumed throughout our modeling framework. Based on prior estimates (36), we assume that 14% of all infections are severe enough to require hospitalization (robustness to alternative rate estimates is shown in *SI Appendix*). While hospitals draw from (and hence average across) areas that are larger than tracts, the heterogeneity shown in Fig. 1 suggests the potential for substantial differences in hospital load over time. Indeed, our models suggest that such differences will occur. Fig. 4 shows the number of patients arriving at each hospital in Seattle and Washington, DC during a typical simulation trajectory. While some hospitals do have demand curves that mirror the city’s overall infection curve, others show very different patterns of demand. In particular, some hospitals experience relatively little demand in the early months of the pandemic, only to be hit hard when infections in the city as a whole are winding down.

Just as hospital load varies, hospital capacities vary as well. As a simple measure of strain on hospital resources, we consider the difference between the number of COVID-19 hospitalizations and the total capacity of the hospital (in beds), truncating at zero when demand outstrips supply. (For ease of interpretation as a measure of strain, we take the difference such that higher values indicate fewer available beds.) Using data on hospital locations and capacities, we show, in Fig. 4, strain on all hospitals in Seattle and Washington, DC during a typical infection trajectory. While some hospitals are hardest hit early on (as would be expected from the aggregate infection curve), others do not peak for several months. Likewise, hospitals proximate to areas of the city with very different infection trajectories experience natural “curve flattening,” with a more distributed load, while those that happen to draw from positively correlated areas experience very sharp increases and declines in demand. These conditions in some cases combine to keep hospitals well under capacity for the duration of the pandemic, while others are overloaded for long stretches of time. These marked differences in strain for hospitals within the same city highlight the potentially complex consequences of heterogeneous diffusion for health care providers.

Looking across cities, we see the same high-variability patterns as observed in Seattle and Washington. In particular, we note that local variation in disease timing leads to a heavy-tailed distribution for the duration at which hospitals will be at capacity. Fig. 4 shows the marginal distribution of hospital overload periods (defined as total number of days at capacity during the pandemic), over the entire sample. While the most common outcome is for hospitals to be stressed for a brief period (not always to the breaking point), a significant fraction of hospitals end up being overloaded for months—or even, in a small fraction of cases, nearly the whole duration of the pandemic.

It should be reiterated that the hospital load model used here is extremely simplified, and that we are employing a no-mitigation scenario. However, these results quite graphically demonstrate that the importance of curve-flattening interventions does not abate once geographical factors are taken into account. On the other hand, these results suggest that differences in hospital load may be substantially more profound than would be anticipated from uniform mixing models, creating logistical challenges and possibly exacerbating existing differences in resource levels across hospitals. At the same time, such heterogeneity implies that resource sharing and patient transfer arrangements could prove more effective as load management strategies than would be suggested by spatially homogeneous models, as hospitals are predicted to vary considerably in the timing of patient demand.

### Social Exposures to Morbidity and Mortality Vary by Location.

In addition to health care strain, the *subjective experience* of the pandemic will potentially differ for individuals residing in different locations. In particular, social exposures to outcomes such as morbidity or mortality may shape individuals’ understandings of the risks posed by COVID-19, and their willingness to undertake protective actions to combat infection. Such exposures may furthermore act as stressors, with potential implications for physical and/or mental health. As a simple measure of social exposure, we consider the question of whether a focal individual (ego) either has experienced a negative outcome themself or has at least one personal contact (alter) who has experienced the outcome in question. (Given the highly salient nature of COVID-19 morbidity and mortality, we focus on the transition to first exposure rather than, e.g., the total number of such exposures, as the first exposure is likely to have the greatest impact on ego’s assessment of the potential severity of the disease.)

**Fig. 5.**
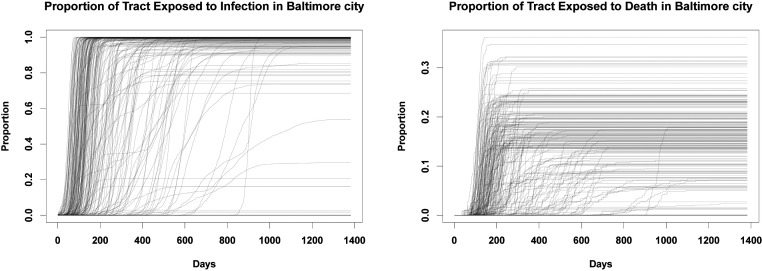
(*Left*) Trajectories showing the fraction of people in each tract in Baltimore who have an infected person in their personal network across time. We see a large degree of spatial heterogeneity, as some tracts are more insulated from others in terms of social exposure. However, by the end of the pandemic, most people across all tracts have been exposed to someone who has had the disease. (*Right*) The fraction of persons in each tract who have an alter who died from COVID-19 in their personal network. On average, only around 20 to 30% of people in any given tract know someone who died, by the end of the pandemic, although this varies widely across tracts.

To examine how social exposure varies by location, we compute the fraction of individuals in each tract who are socially exposed to morbidity or mortality. Fig. 5 shows these proportions for Baltimore, MD, over the course of the pandemic. As with other outcomes examined here, we see considerable variation in timing, with many tracts seeing a rapid increase in exposure to infections, while others go for weeks or months with relatively few persons having a personal contact with the disease. Another notable axis of variation is sharpness. In many tracts, the fraction of individuals with at least one morbid contact transitions from near zero to near one within a matter of days, creating an extremely sharp social transition between the “preexposure world” (in which almost no one present knows someone with the illness) to a “postexposure world” in which almost everyone knows someone with the illness). By contrast, other tracts show a much more gradual increase (sometimes punctuated by jumps), as more and more individuals come to know someone with the disease. In a few tracts that are never hit hard by the pandemic, few people ever have an infected alter; residents of these areas obviously have a very different experience than those of high-prevalence tracts. These distinctions are even more stark for mortality, which takes longer to manifest and which does so much more unevenly. Tracts vary greatly in the fraction of individuals who ultimately lose a personal contact to the disease, and in the rapidity with which that fraction is reached. In many cases, it may take a year or more for this quantity to be realized; until that point, many residents may be skeptical to the notion that the pandemic poses a great risk to them personally.

**Fig. 6.**
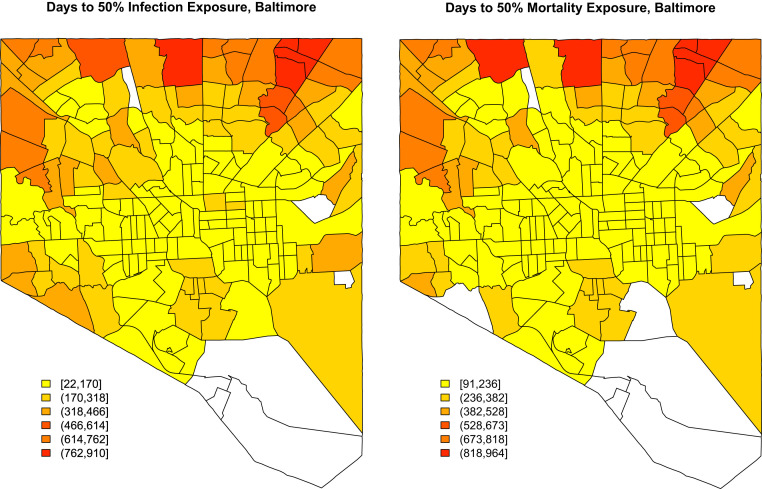
(*Left*) Choropleth showing the time for half of those in each tract to be socially exposed to COVID-19 morbidity in Baltimore, MD. The central parts of the city are exposed far sooner than the northwestern part of the city. (*Right*) Choropleth showing the time for half of those in each tract to be socially exposed to COVID-19 mortality. Central Baltimore is exposed to deaths in personal networks far sooner than the more outlying areas of the city.

By way of assessing the milieu within each tract, it is useful to consider the “cross-over” point at which at least half of the residents who will be socially exposed in a given tract have been socially exposed to either COVID-19 morbidity or mortality. Fig. 6 maps these values for Baltimore, MD. It is immediately apparent that social exposures are more strongly spatially autocorrelated than other outcomes considered here, due to the presence of long-range ties within individuals’ personal networks. Even so, however, we see strong spatial differentiation, with residents in the urban core being exposed to both morbidity and mortality much more quickly than those on the periphery. This suggests that the social experience of the pandemic will be quite different for those in city centers compared to those in more outlying areas, with the latter taking far longer to be exposed to serious consequences of COVID-19. This may manifest in differences in willingness to adopt protective actions, with those in the urban core being more highly motivated to take action (and perhaps resistant to rhetoric downplaying the severity of the disease) than those on the outskirts of the city.

## Discussion

Our simulation results all underscore the potential effects of local spatial heterogeneity on disease spread. The spatial heterogeneity driving these results occurs on a very small scale (i.e., Census blocks), operating well below the level of the city a whole. As the infection spreads, relatively small differences in local network connectivity and the prevalence of bridging ties driven by uneven population distribution can lead to substantial differences in infection timing and severity, leading different areas in each city to have vastly different experiences of the pandemic. Resources will be utilized differently in different areas, as some areas will experience the bulk of their infections far later than others, and the subjective experience of a given individual regarding the pandemic threat may differ substantially from someone in another area. These behaviors are in striking contrast to what is assumed by models based on the assumption of spatially homogeneous mixing, which posit uniform progress of the infection within local areas.

As noted at the outset, our model is based on a no-mitigation scenario, and is not intended to capture the impact of social distancing. While distancing measures by definition limit transmission rates—and will hence slow diffusion—contacts occurring through spatially correlated networks like those modeled here are still likely to show patterns of heterogeneity like those described. One notable observation from our simulations is the long outbreak delay that some census tracts experience, even in the absence of social distancing. This would suggest that relaxation of mitigation measures leading to a resumption of “normal” diffusion may initially appear to have few negative effects, only to lead to deadly outbreaks weeks or months later. Public health messaging may need to stress that apparent lulls in disease progress are not necessarily indicators that the threat has subsided, and that areas “passed over” by past outbreaks could be impacted at any time.

Finally, we stress that conventional diffusion models using locally homogeneous mixing have been of considerable value in both pandemic planning and scenario evaluation. Our findings should not be taken as an argument against the use of such models. However, the observation that incorporating geographical heterogeneity in contact rates leads to radically different local behavior would seem to suggest that there is value in including such effects in models intended to capture outcomes at the city or county level. Since these are the scales on which decisions regarding infrastructure management, health care logistics, and other policies are often made, improved geographical realism could potentially have a substantial impact on our ability to reduce lives lost to the COVID-19 pandemic.

## Supplementary Material

Supplementary File

## Data Availability

Social networks and analysis code data have been deposited in Harvard Dataverse (https://doi.org/10.7910/DVN/B9XKSR) (37).
