# Supplementary material for: Spatial heterogeneity can lead to substantial local variations in COVID-19 timing and severity
Source: Proc Natl Acad Sci U S A. 2020 Sep 10;117(39):24180–7. doi: 10.1073/pnas.2011656117 (PMC7533653; doi:10.1073/pnas.2011656117)
Supplement: Supplementary File [file pnas.2011656117.sapp.pdf]

Supplementary Information for

Spatial Heterogeneity Can Lead to Substantial Local Variations in COVID-19 Timing and Severity

Loring J. Thomas, Peng Huang, Fan Yin, Xiaoshuang Iris Luo, Zack W. Almquist, John R. Hipp, Carter T. Butts

Carter T. Butts.
E-mail: buttsc@uci.edu

This PDF file includes:

Supplementary text
Figs. S1 to S5
Tables S1 to S2
SI References

Supporting Information Text

Introduction

In this supplement, we go into more depth on Spatial Interaction Functions, Spatial Bernoulli Models, the setup and parameterization of our simulations, and the parameter estimates that were used for this paper. We also provide additional analyses regarding the role of local first-passage times as determinants of individuals' waiting times to infection, and the impact of alternative estimates of hospitalization rates on numbers of days at which hospitals would be expected to be at capacity.

Spatial Interaction Function

A Spatial Interaction Function (SIF) describes the marginal probability of a tie between any two nodes, given the distance between them. We denote the SIF by $\mathcal{F}(\mathcal{D}_{ij}, \theta)$, with $\mathcal{D}_{ij}$ being the distance between vertices i and j, and θ being the parameters for the function. Prior literature has found that spatial interaction functions for social networks like those of interest here tend to be of the power law or attenuated power law form (1). Following this, we employ SIFs of the form $\mathcal{F}(\mathcal{D}_{ij}, \theta) = \frac{p_b}{(1+\alpha\mathcal{D}_{ij})^{\gamma}}$. Here, p_b represents the base tie probability, which can be thought of as the probability of a tie between two individuals residing at the same location. α is a scaling parameter that determines the phenomenological unit of distance for the decay in tie probability, and γ is the parameter that determines the weight of the tail (higher values imply fewer long-range ties, *ceteris paribus*).

We employ two SIFs in this paper, using models for social interactions and face-to-face interactions employed in prior studies (2, 3). The social interaction SIF declines with a γ of 2.788, while the face-to-face SIF declines with γ of 6.437. The parameters for the social interaction SIF are $p_b = 0.533$, $\alpha = 0.032$, $\gamma = 2.788$, and the parameters for the face-to-face SIF are $p_b = 0.859$,$\alpha = 0.035$, $\gamma = 6.437$ (3).

Spatial Bernoulli Models

The Bernoulli Network Models are a class of random graph models in which each edge occurs as a Bernoulli trial, possibly with a distinct probability of occurrence. In a spatial Bernoulli graph, tie probabilities are determined by a Spatial Interaction Function, applied to the pairwise distances between individuals within some space (here, geographically determined using Census data). Spatial Bernoulli models are highly scalable due to the conditional independence of edges, but allow for extremely complex structure due to the heterogeneity in edge probabilities induced by the SIF; likewise, they naturally produce properties such as local cohesion and degree heterogeneity observed in many types of social networks (2). Formally, we can specify a Spatial Bernoulli Model by $\Pr(Y_{ij} = 1) = \mathcal{F}(\mathcal{D}_{ij}, \theta)$, where Y_{ij} is a dichotomous indicator for the presence of the i, j edge, and $\mathcal{F}(\mathcal{D}_{ij}, \theta)$ is a Spatial Interaction Function taking as inputs the i, j distance $\mathcal{D}_{ij}$ and parameters θ.

Network Simulations

To simulate diffusion of COVID-19, we require a contact network. Here, we employ the above-described spatial Bernoulli graphs, with node locations for each of our 19 study locations drawn based on block-level Census data (including clustering within households, an important factor in disease diffusion). We follow the protocols described in (2, 4) to generate node positions, specifically using the quasirandom (Halton) placement algorithm. Node placement begins with the households in each census block, using Census 2010 data with regions defined per (3). The quasirandom placement algorithm uses a Halton sequence to place households in space within the areal unit in which they reside. If any two households are placed within a critical radius of each other, then the algorithm "stacks" the households on top of each other by introducing artificial elevation (simulating e.g. a multistory apartment building). Once all households are placed, individuals within households are placed at jittered locations about the household centroid. (Individuals not otherwise attached to households are treated as households of size 1.)

Given an assignment of individuals to spatial locations, we simulate spatial Bernoulli graphs using the models specified above. We generate two networks for each city, one with the social interaction SIF, and the other with the face-to-face interaction SIF. To form a network of potential high-risk contacts, we then merge these networks (which share the same node set) by taking their union, leading to a network in which two individuals are tied if they either have an ongoing social relationship or would be likely to have extensive face-to-face interactions for other reasons (e.g., interacting with neighbors). This process is performed for each city in our sample.

List of Cities. Table S1 lists the cities that we use for our simulations. These data are drawn from (3), with population data updated to reflect the most recent (2010) decennial Census.

Disease Simulations

We conduct a series of simulations to examine the spread of COVID-19 across city-sized networks. These simulations use a simple continuous-time network diffusion process, the general description of which are described in the main text. The input for the diffusion simulation is a network and a vector of initial disease states (*susceptible*, *latent* (infected but not yet infectious), *infectious*, *recovered*, and *deceased*), and the output is detailed history of the diffusion process up to the point at which a steady state is obtained (i.e., no infectious individuals remain). Infection occurs via the network, with currently infectious individuals

Table S1. List of study communities.

	City/County
1	Buffalo
2	Baltimore
3	Cincinnati
4	Cleveland
5	Denver
6	Indianapolis
7	Miami
8	Milwaukee
9	Nashville
10	Pittsburgh
11	Rochester
12	Sacramento
13	Salinas
14	San Diego City
15	Seattle
16	St. Petersburg
17	Tampa
18	Tuscon
19	Washington DC

infecting susceptible alters as Poisson events with a fixed rate. The transitions between latent and infectious, and infectious and either recovery or mortality are governed by gamma distributions estimated from epidemiological data. Table S2 shows the estimated shape and scale parameters for the gamma distributions employed here. The parameters for waiting time to infectiousness are directly available in the Appendix of (5), while those for the recovery and death are estimated by matching the mean and standard deviation of durations reported in the literature (6). Selection into death versus recovery was made via a Bernoulli trial drawn at time of infection (thereby determining which waiting time distribution was used), with the estimated mortality probability being 0.0138 using the case fatality rate adjusted for under-ascertainment reported in (6). Under these parameters, the median time to infectiousness is 5.2 days, with 95% of cases falling between 2 and 10.1 days; once infectious, respective median times to recovery and death are 25.1 days (95% range 9.6 to 52.2) and 16.9 days (95% range 5.5 to 38.3). We note in particular that these distributions incorporate the clinically observed skewness in recovery times, with many cases resolving in less than two weeks but a non-trivial fraction persisting for six weeks or longer.

Table S2. Shape and Scale parameters for Gamma distributions for durations (unit: day).

	Death	Recovery	Infectious
Shape	4.566	5.834	5.807
Rate	0.251	0.219	1.055
Scale (i.e., 1/Rate)	3.984	4.566	0.948

Infection Rate Parameter Estimation

To determine the infection rate (the only free parameter for the models used in our simulations), we simulate the diffusion of virus in Seattle and fit it to the over-time death rate of the King County, WA before the first shelter-in-place order went into effect on March 23, 2020. We limit our data to this time period because our simulation employs a no-mitigation scenario. A grid search strategy was employed to determine the expected days to transmission (which is the inverse of infection rate), and the number of days between the existence of the first infected cases and the first confirmed cases (aka the time lag, a nuisance parameter that is relevant only for estimation of the infection rate). The time lag is treated as an integer and the expected days to transmission as a continuous variable. For each lag/rate pair, we randomly take 5 draws from the expected infection waiting time distribution, add them to the lag time (i.e. the introduction of the true patient zero for the initial outbreak), and simulate 50 realizations of the diffusion process (redrawing the network each time). The diffusion rate parameter was selected based on minimizing the mean squared error between the simulated death rate and the observed death rate over the selected period. The first round of grid-search divided the expected days of search into 100 intervals, from (0,1) to (99,100), with days of lag ranging from 1 to 100 days. The second round of grid-search, based on the performance of the first round, divided the expected days of search into 240 intervals, from (40.00,40.25) to (99.75,100.00), with days of lag ranging from 1 to 60 days. The grid-search suggests that the expected days to transmission is 82.875 (82.75,83.00) days (Fig S1); that is, in a hypothetical scenario in which a single infective ego remained indefinitely in the infective state, and a single alter remained otherwise susceptible, the average waiting time for ego to infect alter would be approximately 80 days. While this may at first blush appear to be a long delay, it should be borne in mind that this embodies the reality that no individual is likely to infect any *given* alter within a short period (since, indeed, ego and alter may not happen to interact within a narrow window). With many alters, however, the chance of passing on the disease is quite high. Likewise, we note that the thought experiment

above should not be taken to imply that actors remain infectious for such an extended period of time; per the above-cited epidemiological data, individuals typically remain infectious for roughly 25.1 days (95% range 9.6-52.2). When both delay times are considered, the net probability of infecting any given alter prior to recovering is approximately 27%. We further calculated the corresponding basic reproductive number (R_0), which is the product of the probability of infection (27%) and the mean degree of the networks (10). The corresponding basic reproductive number in the diffusion simulation model is 2.7, compatible with estimates of R_0 at the pre-mitigation stage in other literature (7–9).

Simulation Replicates

To supplement the results on the variation in the peak infection time given in the main text, we ran a series of simulation replicates. Figure 3 in the main text shows the data from the figure below aggregated across all replicates. In the supplemental Figure S2, we break out the peak infection days in each city, by replicate. These data show that the significant variation in Figure 3 is not due to the number of replicates that were run, but instead due to the intrinsic variation that is present (i.e., spatial heterogeneity).

Timing and Shape of Infection Curves

While our simulations show substantial heterogeneity in infection patterns across tracts, it may be hypothesized that this pattern is driven by a characteristic pattern of infection *within tracts*, with the heterogeneity being driven by the differences in *arrival time* of the infection to different tracts. (Such a pattern has been hypothesized by e.g. (10) at the national level to explain heterogeneity at global scales.) To examine this possibility, we ran several additional analyses. First, to assess whether diffusion patterns within tracts follow a *universal curve*, we compare standardized within-tract prevalence curves across the entire sample of cities. To standardize the infection curves, we apply the following transformations. First, all selected infection prevalence curves (i.e., active cases as a function of time) were translated to the origin, such that the first infection occurs at time 0. Next, we standardized the maximum prevalence by dividing the number of infections active on any day by the maximum prevalence for the tract. Finally, we standardized the time scale of the prevalence curve so that the mean prevalence time (i.e., the centroid of the temporal distribution) occurs at unit time. The resulting standardized curves reflect the pure shape of the infection trajectory; if infection follows a universal pattern at local scales (up to an affine transformation), then the standardized curves should be approximately identical.

To assess the standardized curves, we chose a stratified sample of tracts from across the sample, selecting 5 tracts at random from each city. To avoid artifacts from tracts with insufficient infections, we exclude tracts with fewer than 20 infections during the time course of the simulation. Figure S3 shows the resulting distribution of standardized prevalence curves. In line with the universality hypothesis, there is a dominant functional form approximately followed by a large fraction of tracts. However, we also observe a large fraction of tracts that deviate from this central pattern. Modes of deviation are idiosyncratic, and include multi-modality, substantial differences in skewness, and differences in "roughness" over time. Quantitatively, relatively large variations in standardized time to maximum prevalence are observed, with maxima for the majority of the sample falling between approximately 0.5 and 1.5 time-to-mean-prevalence units. These observations suggest that, while there is a fairly *common* tract-level infection pattern, this pattern coexists with a wide range of other types of trajectories and is better thought of as a central tendency than a universal phenomenon.

Even if there is not a universal tract-level diffusion curve, it could still be the case that the majority of heterogeneity in infection times could be explained by the time taken for the infection to reach each tract: in particular, if the city-level diffusion process reflects a union of very small outbreaks with the waiting time for the infection to "jump" from one area to another being large compared to the time needed for the local outbreaks to reach all susceptibles within the local area, then the primary determinant of individual infection time would be the time taken for the infection to reach the individual's local region. We examine this hypothesis in figure S4. The left-most boxplot of figure S4 shows the distribution of times to infection for all individuals in our sample, timed from the onset of the first infection. The right-most boxplot shows the corresponding waiting times for the same individuals, *net of the first appearance of the infection in their tracts.* If infection time were determined primarily by the time it takes for the infection to reach an individual's local area, we would see a substantially compressed distribution relative to the total waiting time; on the contrary, the two are nearly the same, falsifying the conjecture that diffusion to the local area is the driver of waiting time heterogeneity.

The reason for this lack of compression can be seen in the middle boxplot of figure S4, which shows the distribution of waiting times for the first case in each tract. We can see that, while individual infection times vary markedly, first passage times to tracts are both short and highly compressed: once introduced, the infection diffuses rapidly to nearly all tracts. Further, we can see that nearly all tracts are reached long before the majority of individuals are infected – thus, diffusion to local areas is not the primary limiting factor determining individual waiting times. Instead, the relative permeability of those areas to diffusion (which may depend upon factors such as local population density and the presence of barriers to interaction) appears to play a much greater role in governing the distribution of infection times.

Robustness of Spatial Heterogeneity on Hospital Load

Currently, considerable uncertainty exists regarding the fraction of SARS-CoV-2 infections leading to hospitalization; as such, it is useful to verify that the overall patterns of spatial heterogeneity seen here are robust to the hospitalization rate. On the high end, the World Health Organization and the CDC have estimated the rate to be 20% and 20-30%, respectively, in the absence

of mitigation (11, 12). Arguably, these estimates are inflated by under-reporting of asymptomatic or mildly symptomatic infections, particularly given the poor state of testing in the early stage of the pandemic. At the opposite extreme, one recent serology study contended that less than 10% of infections were reported in USA (13), implying a potential hospitalization rate in the neighborhood of 2%. This study, too, faces problems with selection, as population prevalence was based on a convenience sample from patients seeking health care during the pandemic (and who are hence disproportionately likely to be infected); the true extent of under-reporting is thus likely to be smaller than this estimate (and the hospitalization rate correspondingly higher). Taking these two estimates as upper and lower bounds on the likely rate of COVID-19 hospitalizations, we replicated our analysis on hospital load using 20% and 2% (i.e., 10% of 20%) as respective probabilities of hospitalization per infection.

Fig. S5 shows the respective marginal distributions of hospital overload periods with 20% and 2% hospitalization rates. While the scale of full-capacity days varies with the selection of the hospitalization rate, the shape of these distributions closely resembles that of Fig. 7 in the main text: despite the majority of hospitals running at capacity for a relatively short period of time, a sizable fraction of hospitals experience overloads for very long periods. The persistence of this pattern over a full order of magnitude variation in hospitalization rates demonstrates the potential of unmitigated COVID-19 infections to severely strain local resources even under fairly optimistic scenarios, and suggests that substantial inequalities in healthcare service demand are robust to detailed hospitalization rates. However, we also note that the current level of uncertainty in hospitalization rates serves as a significant obstacle to quantitative prediction of the spatial distribution of hospital load for planning or response purposes. Particularly given the large gap between anticipated load on "typical" units and those expected to be hit hardest, more refined rate estimates would seem to have the potential to inform important resource allocation decisions.

Code and Data Availability

Code and data needed to replicate the simulation and analysis for this paper can be found at:

Loring J. Thomas; Peng Huang; Fan Yin; Xiaoshuang Iris Luo; Zack W. Almquist; John R. Hipp; Carter T. Butts, 2020, "Replication Data for: Spatial Heterogeneity Can Lead to Substantial Local Variations in COVID-19 Timing and Severity," `https://doi.org/10.7910/DVN/B9XKSR`, Harvard Dataverse.

References

1. Butts CT, Acton RM (2011) Spatial modeling of social networks. *The Sage Handbook of GIS and Society Research. Thousand Oaks, CA: SAGE Publications* pp. 222–250.
2. Butts CT, Acton RM, Hipp JR, Nagle NN (2012) Geographical variability and network structure. *Social Networks* 34(1):82–100.
3. Hipp JR, Butts CT, Acton R, Nagle NN, Boessen A (2013) Extrapolative simulation of neighborhood networks based on population spatial distribution: Do they predict crime? *Social Networks* 35(4):614–625.
4. Almquist ZW, Butts CT (2012) Point process models for household distributions within small areal units. *Demographic Research* 26:593–632.
5. Lauer SA, et al. (2020) The incubation period of coronavirus disease 2019 (COVID-19) from publicly reported confirmed cases: estimation and application. *Annals of Internal Medicine*.
6. Verity R, et al. (2020) Estimates of the severity of coronavirus disease 2019: a model-based analysis. *The Lancet Infectious Diseases*.
7. Li R, et al. (2020) Substantial undocumented infection facilitates the rapid dissemination of novel coronavirus (SARS-CoV-2). *Science* 368(6490):489–493.
8. Li Q, et al. (2020) Early transmission dynamics in Wuhan, China, of novel coronavirus–infected pneumonia. *New England Journal of Medicine* 382(13):1199–1207.
9. Wu JT, Leung K, Leung GM (2020) Nowcasting and forecasting the potential domestic and international spread of the 2019-nCoV outbreak originating in Wuhan, China: a modelling study. *The Lancet* 395(10225):689–697.
10. Brockmann D, Helbing D (2013) The hidden geometry of complex, network-driven contagion phenomena. *Science* 342(6164):1337–1342.
11. CDC COVID and Response Team (2020) Severe outcomes among patients with coronavirus disease 2019 (COVID-19)—United States, February 12–March 16, 2020. *MMWR Morb Mortal Wkly Rep* 69(12):343–346.
12. World Health Organization (2020) *Media Statement: Knowing the risks for COVID-19*.
13. Havers FP, et al. (2020) Seroprevalence of antibodies to SARS-CoV-2 in 10 sites in the United States, March 23-May 12, 2020. *JAMA Internal Medicine*.

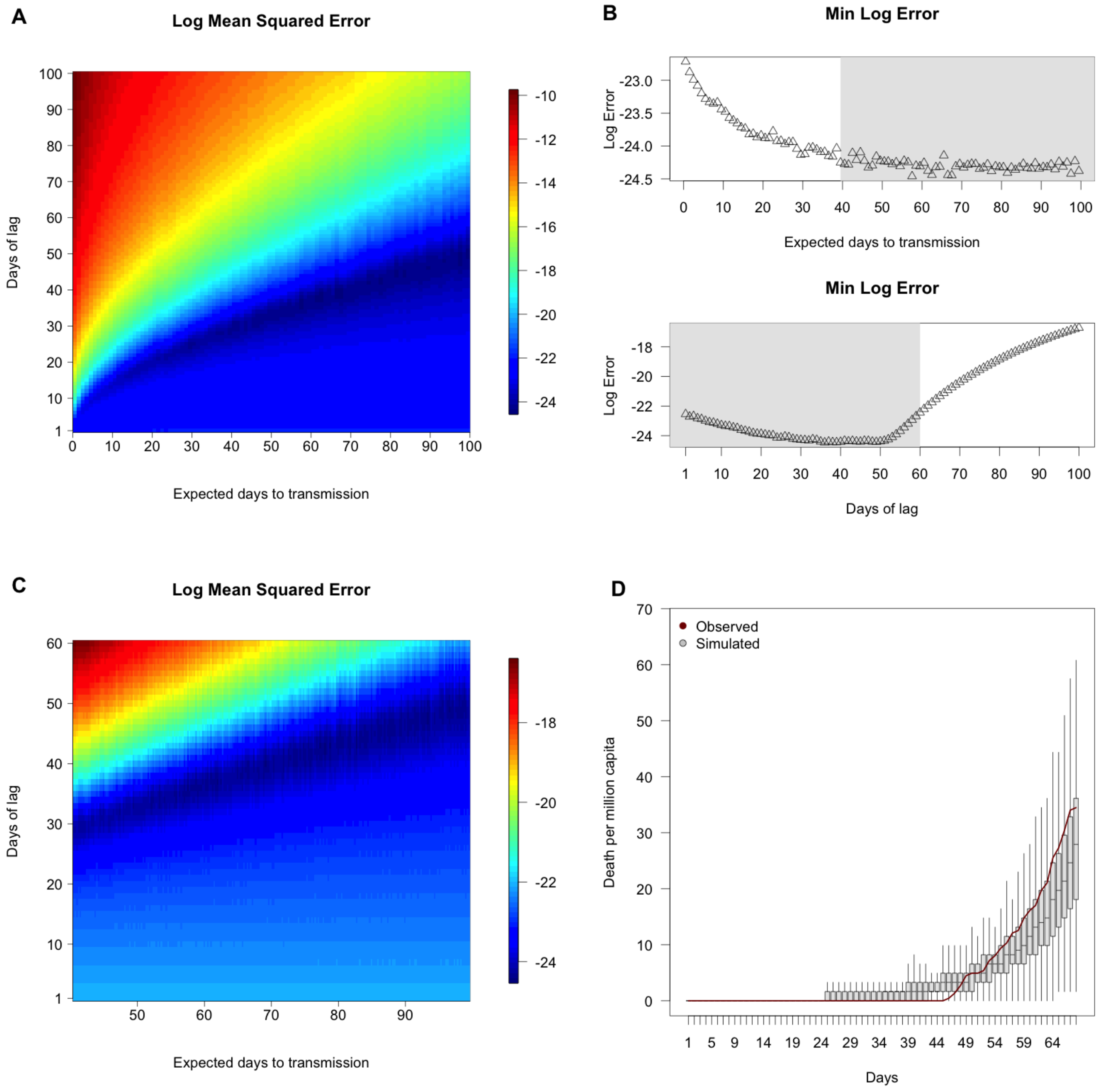

Fig. S1. Grid-search for infection rate. The mean squared error (in logarithm form) for simulations with combinations of days of lag and expected days to transmission, for the first round (**A**) and the second round (**C**). The cross-sectional analysis of the minimum log error for each days of lag and expected days to transmission (**B**) suggests the interval of both variables for the second round of search (areas in gray). Curves of death rate based on the best-fit parameter: 44 days of lag, 82.875 expected days to transmission (**D**).

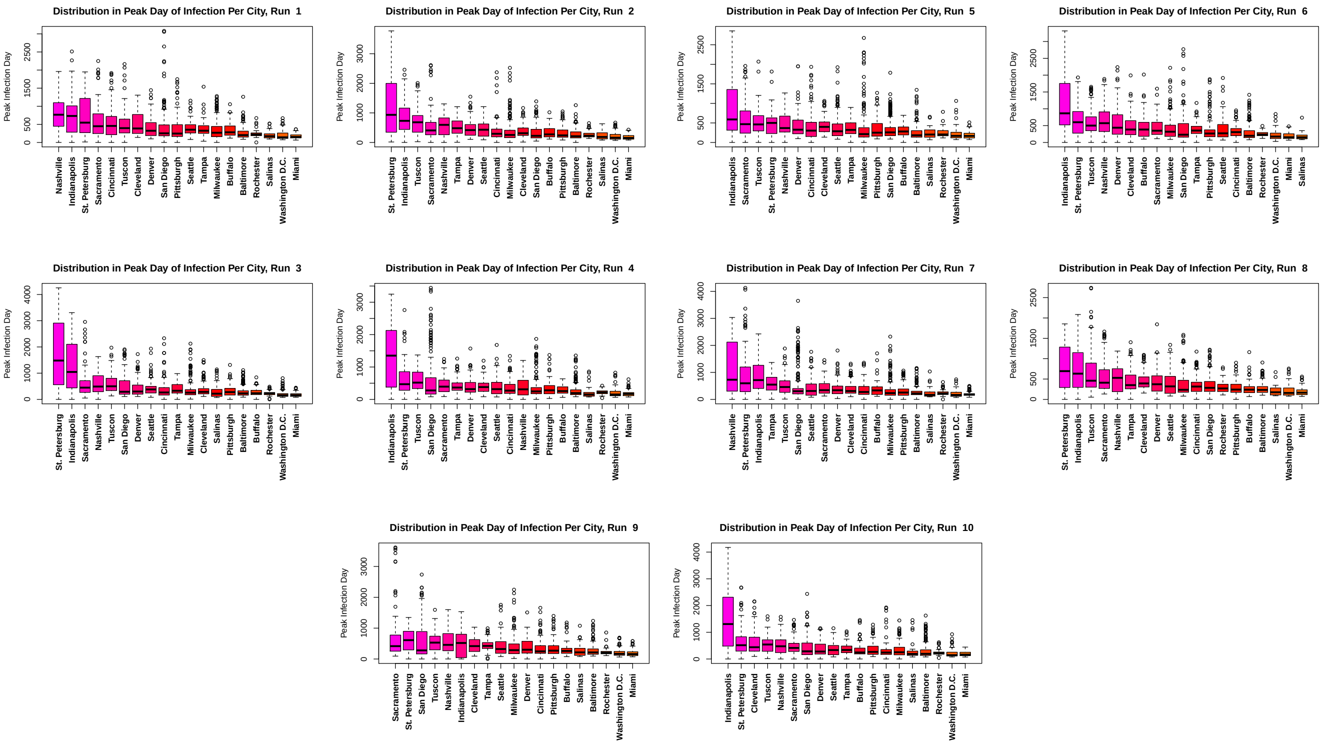

Fig. S2. Boxplot showing the peak infection days across 10 replicates for each city in the sample. There is a large degree of heterogeneity within each city, showing that the day that the infection peaks for any given tract is not uniform at all. Within any given city, there is a consistently high amount of variance in the peak infection day. In other words, the variance that we show here is a property of the spread of the disease, rather than the number of simulation replicates.

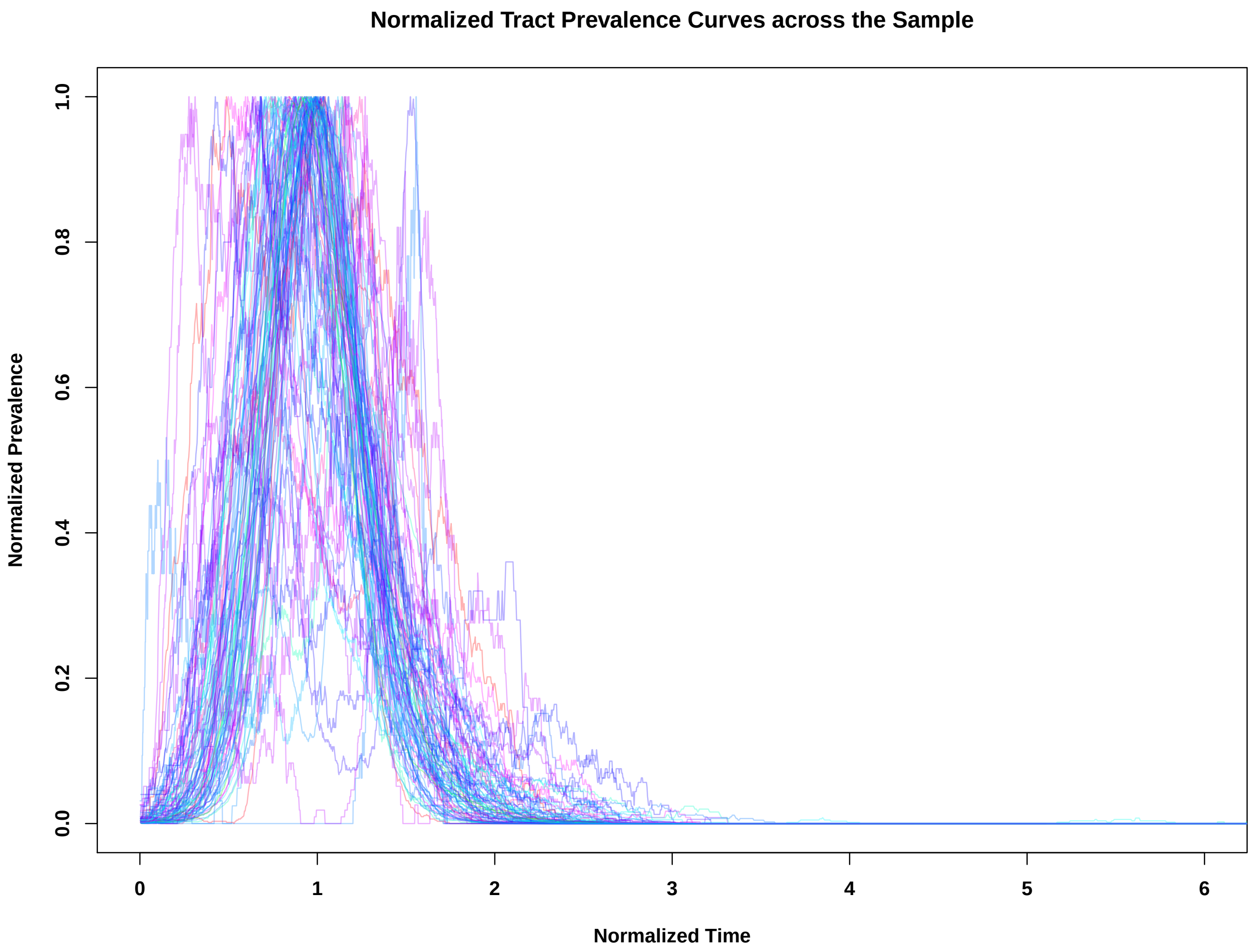

Fig. S3. Standardized infection curves at the tract level. Many tracts approximately follow the stereotypical “bell-shaped” pattern, but a large number of tracts deviate by being irregular, multimodal, and/or long-tailed. (Note: all trajectories scaled to unit maximum; apparent truncation actually reflects between-curve variation in time to maximum prevalence.)

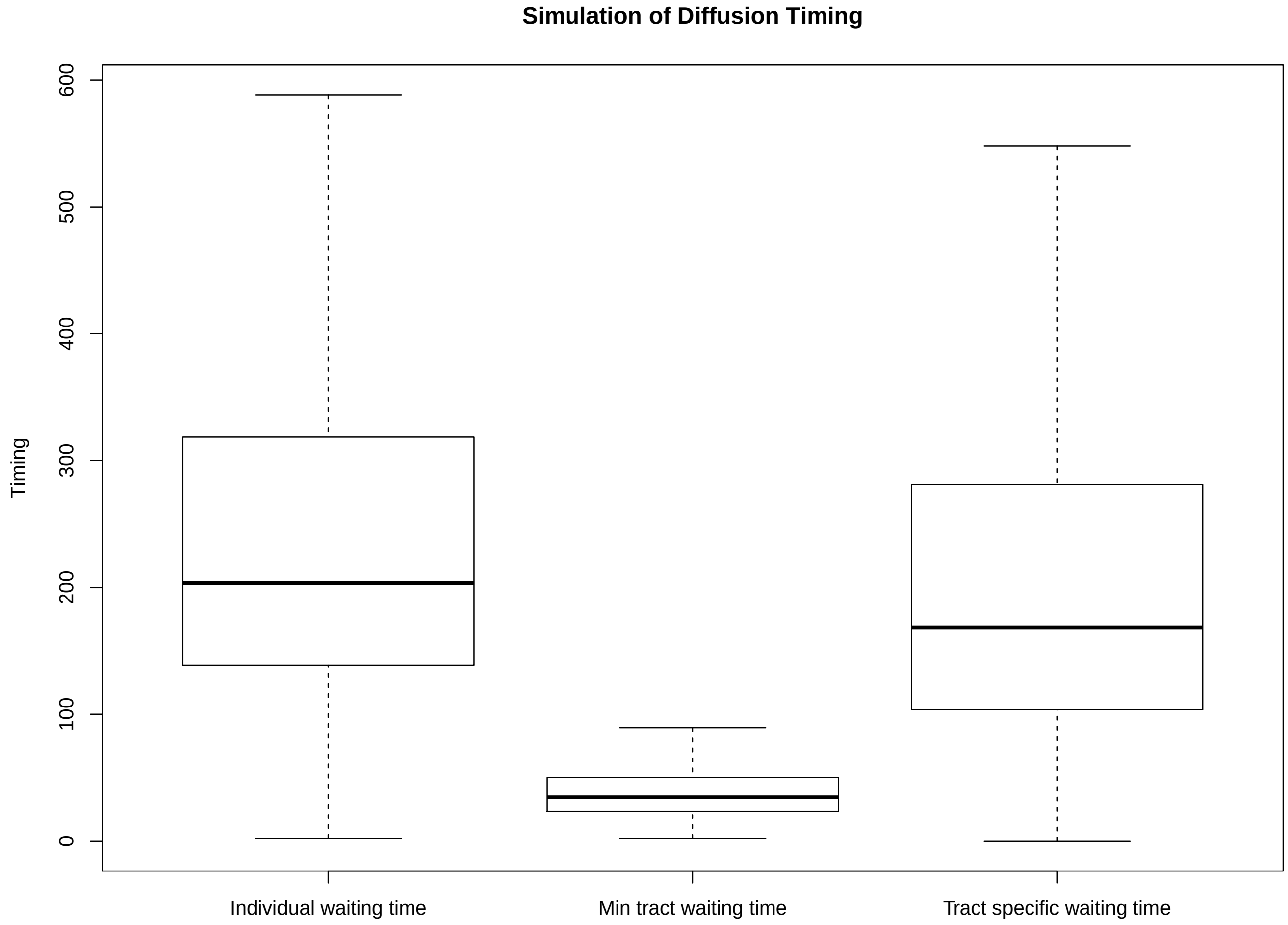

Fig. S4. Respective distributions of individual infection times (left), tract arrival times (middle), and tract specific infection times (right) for the tract sample. Individual infection times relative to arrival within tracts show little difference from infection times relative to the start of the larger outbreak, as tract arrival times are substantially shorter than the time needed to diffuse within tracts.

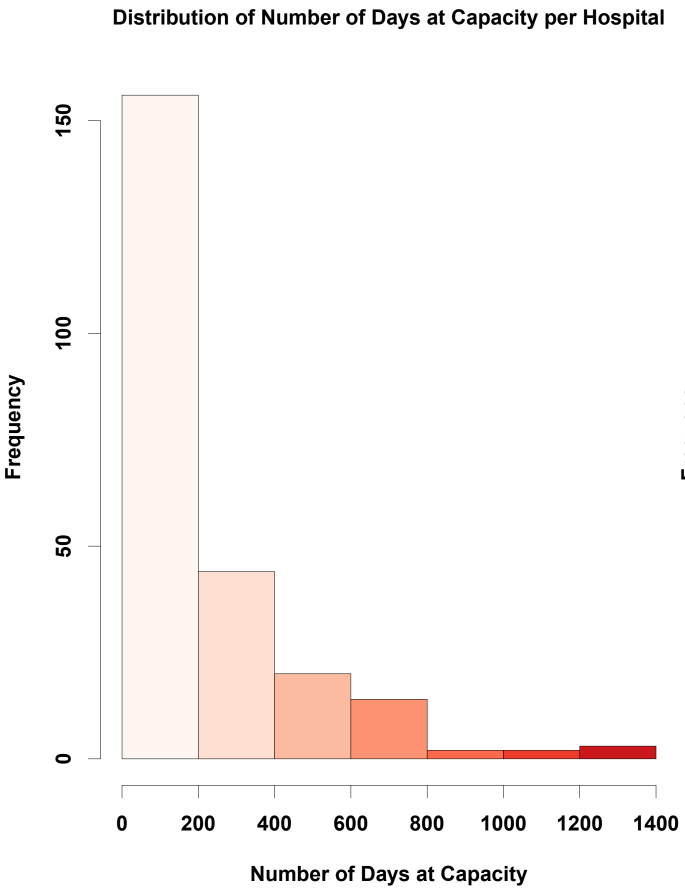

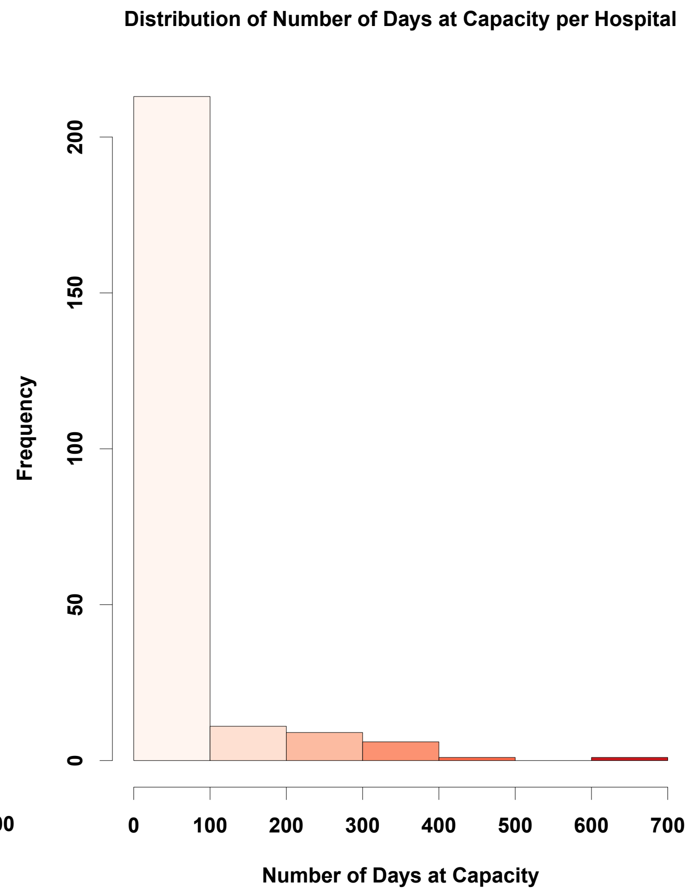

Fig. S5. (Left) The distribution of days that hospitals are at capacity, with a 20% hospitalization rate. (Right) The distribution of days that hospitals are at capacity with a 2% hospitalization rate. While higher hospitalization rates lead to higher levels of strain overall, extreme inequality in load persists in both scenarios.
